# Impact of Cell Wall Composition on Maize Resistance to Pests and Diseases

**DOI:** 10.3390/ijms14046960

**Published:** 2013-03-27

**Authors:** Rogelio Santiago, Jaime Barros-Rios, Rosa A. Malvar

**Affiliations:** Misión Biológica de Galicia (CSIC), Pontevedra E-36080, Spain; E-Mails: jaime.barros-rios@plantphys.umu.se (J.B.-R.); rmalvar@mbg.csic.es (R.A.M.)

**Keywords:** maize (*Zea mays* L.), pests and diseases, cell wall, cellulose, arabinoxylans, hydroxycinnamates, lignin, ferulic acid, borer, maize weevil, ear and stalk rot, rust, leaf blight

## Abstract

In cereals, the primary cell wall is built of a skeleton of cellulosic microfibrils embedded in a matrix of hemicelluloses and smaller amounts of pectins, glycoproteins and hydroxycinnamates. Later, during secondary wall development, *p-*coumaryl, coniferyl and sinapyl alcohols are copolymerized to form mixed lignins. Several of these cell wall components show a determinative role in maize resistance to pest and diseases. However, defense mechanisms are very complex and vary among the same plant species, different tissues or even the same tissue at different developmental stages. Thus, it is important to highlight that the role of the cell wall components needs to be tested in diverse genotypes and specific tissues where the feeding or attacking by the pathogen takes place. Understanding the role of cell wall constituents as defense mechanisms may allow modifications of crops to withstand pests and diseases.

## 1. Introduction

**Figure 1:**
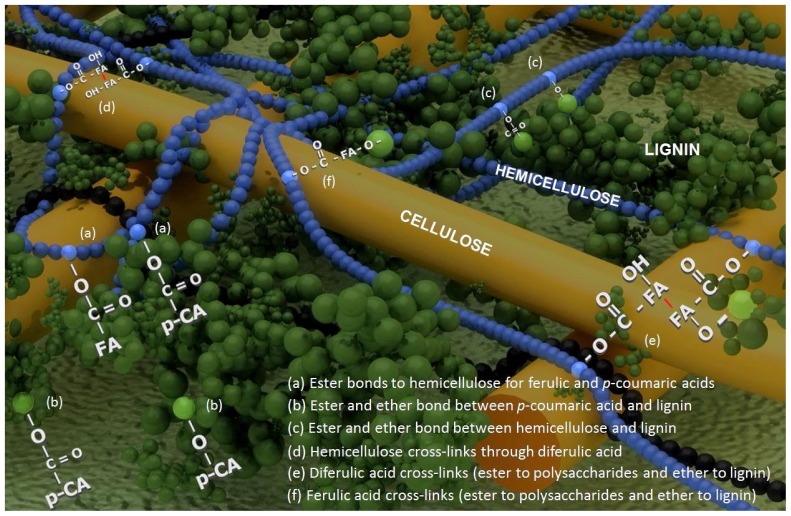
Representation of the secondary cell wall structure in maize. Adapted from Barros-Rios *et al*. [10].

Plants rely on their cell walls to provide shape and strength to cells, to glue cells together and to give rigidity to the whole plant [1]. The composition of plant cell walls varies significantly from one cell type to another, one species to another, and between accessions within species [2]. There is a special overview of the structure and synthesis of plant cell walls in Carpita and McCann, 2000 [3]. The growing cell wall consists mainly of cellulose microfibrils embedded in a matrix of hemicelluloses, pectins, proteins and phenolics [3,4]. For instance, a typical dicot wall contains 30% cellulose, 30% hemicellulose, 35% pectin and 1%–5% structural protein. By contrast, a typical grass species contains, 25% cellulose, 55% hemicellulose (arabinoxylans, xyloglucan and, in some tissues, mixed-linked glucans) and only 10% pectin [5]. The xylans are substituted with arabinose and glucuronic acid side chains, and are called arabinoxylan (AX) or glucuronoarabinoxylans (GAX) [6]. A proportion of the arabinose side chains are further substituted with phenolic acid residues, notably ferulic (4-hydroxy-3-methoxycinnamate), *p*-coumaric (4-hydroxycinnamate) and 4-*O*-methylglucuronic acids [7]. The arabinoxylans in grasses serve a major structural role by binding to cellulose microfibrils and becoming oxidatively cross-linked with each other and with lignin by phenolics [8,9]. Later in plant development, the lignin polymer becomes a significant constituent of the cell wall (Figure 1).

Plants anti-nutritional defenses against insects can occur as both pre-ingestion to limit food supply, and post-ingestion to reduce nutrient value to the attacking insect. In the pre-ingestion phase, host plants can limit food supplies to insects via physical barriers such as the cell wall fortification. Lignin and other phenolics can strengthen cell walls against digestion and therefore can be anti-nutritional [48,49]. Plant cell wall components are important factors dictating plant resistant to mechanical injury and the tearing action of mandibles [50,51]. On the other hand, diverse cell wall components have been implicated in disease resistance by a number of researchers [52–55]. Lignin and the presence of phenols in host cell walls act as a physical barrier against fungal penetration [55,56]. In the current review, we describe the role of key cell wall components as barriers that limit insects feeding and diseases progression (Table 1). The research is focused on the maize (*Zea mays* L.) cultivation as a model of monocotyledon crop.

## 2. Cell Wall Polysaccharides

Cellulose is the most abundant polymer in nature. It is the main component of the plant cell wall, highly stable and insoluble in water, is synthesized by plasma membrane protein complexes, and is deposited directly on the wall in a directional way [57]. It is a linear homopolymer composed of β (1→4) linked d-glucose units. Cellobiose [glucose dimer linked by β (1→4)] is the repeating unit of cellulose. In contrast, the term hemicellulose refers to a diverse class of polysaccharides which differ from the cellulose because this fraction is amorphous and easily soluble or hydrolysable in alkaline or acid solutions. Hemicellulose is tangled with cellulose microfibrils by hydrogen bonds giving the latter a greater inaccessibility [58]. In maize the most abundant polysaccharides are GAX, considered substitutes of pectins [59], and β-glucans with mixed connections. The basic structure of GAX is a β (1→4)-d-xylan backbone with substitutions of arabinose, glucuronic acid and acetic acid.

Most of the literature on the role of cell wall polysaccharides in pests and diseases resistance is based on the study of fibers. Fiber is composed largely of cellulose, hemicellulose and lignin, which are the primary components of plant cell walls [60]. Neutral detergent fiber (NDF) is the most common measure of fiber; it covers most of the structural components in the cell walls (*i.e.*, cellulose, hemicellulose and lignin). On the other hand, acid detergent fiber (ADF) refers solely to the insoluble fiber within a plant cell (cellulose and lignin). In this section we present research concerning the fibers as a bulk (including acid detergent lignin (ADL)), while a more specific role of the lignin polymer in resistance is discussed in section 4.

Fibers can disturb feeding insects from both the nutritional and physical point of view. High levels of fiber can increase the bulk density of the diet, and thereby, larvae are unable to ingest adequate amounts of nutrients and water. Besides, tissues with high fiber content are tougher and more resistant to the cutting or chewing action of the mandibles. In addition, some studies pointed out the role of cell walls polysaccharides as barriers to the fungal invasion.

European corn borer (ECB), *Ostrinia nubilalis*, Hübner, is a tunneling insect that bores into maize stems causing physical damage, disruption of nutrient and water flow, stalk lodging and grain damage, leading to extensive yield losses. In temperate regions it has one to three generations per year. Damage to the plant results from leaf feeding in the whorl (first generation), leaf sheath, tassel and stalk tunneling (first, second and third generations), and ear damage (second and third generations). Whorl, sheath and collar tissues of 22 inbred lines and 3 full-sib families were evaluated for first and second generation ECB damage, and ADF, lignin and silica concentration were quantified [11]. For the first generation ECB the role of lignin in the resistance was shown, while for the second generation ECB the silica was the only significant trait related with resistance.

This initial study was focused on the analysis of diverse genotypes, but it is interesting to analyze the relationships between the cell wall polysaccharides and resistance within the same gene pool. Buendgen and coauthors [13] studied if five cycles of recurrent selection for first and second generation ECB resistance in the BS9 maize population resulted in correlated changes in ADF, NDF, cellulose, hemicellulose, lignin, ash, and N concentration in the whorls, leaf sheaths, and stalks. No appreciable changes in NDF, ADF, lignin, ash, or N concentrations in the whorls were associated with the first generation ECB resistance. Traits like the hydroxamic acid 2,4-dihydroxy-7-methoxy-1,4- benzoxazin-3-one (DIMBOA) were more effective on this relationship [19,61]. However, the authors highlight the highly significant linear responses observed in the leaf sheath for NDF, ADF, cellulose, and lignin over cycles of selection in BS9, suggesting a genetic association of these constituents with the second generation ECB resistance. Similar results were obtained when stalk tissues were analyzed. Furthermore, Bergvinson and coworkers [19], within the same recurrent selection material, concluded that feeding on mature leaf, leaf sheath, and stalk tissues appears to be affected, among other factors, by fiber.

Two maize populations, WFISIHI and WFISILO, which were developed for high and low concentrations, respectively, of indigestible leaf-sheath constituents (ADF, lignin, and silica) were evaluated by Buendgen *et al*. [13] in relation to ECB resistance. The comparison of tissue composition between WFISIHI and WFISILO suggests that, contrary to the results in BS9, structural carbohydrate, lignin, and ash appeared to have little effect on ECB resistance. As the authors remark, the genetic backgrounds of BS9, WFISIHI, and WFISILO differ greatly. Resistance mechanisms in maize can differ depending on genotype analyzed [62]. Subsequently, Ostrander and Coors [12] found that after two cycles of selection WFISILO and WFISIHI have diverged further for all compositional traits except whorl ADF, and WFISILO C2 was significantly more susceptible to first and second generation ECB infestations than WFISIHI C2. In the same way, selection for high NDF and lignin concentrations in BS9 C2 produced materials with greater second generation ECB resistance than selection for low concentration. New evidence of the relationship between the fibers and the ECB second generation resistance was shown in a study conducted by Beeghly and coauthors [14]. Significant phenotypic and genetic correlations were found between ECB tunneling and fiber components. Overall, we can note an association between the plant cell wall composition and ECB resistance (mainly in the second generation), although the genetic backgrounds influence this relationship.

In an incidental way, Martin *et al*. [15] carried out a divergent selection program for stalk strength by using a rind penetrometer. After twelve cycles of divergent selection stalk strength was decreased and increased in the high and low directions of selection, respectively. Selection for high rind penetrometer resistance was effective at providing resistance to second-generation ECB damage as well as resistance to stalk lodging. Interestingly, crude fiber, cellulose and lignin concentration increased in the high direction of selection, suggesting that stalk composition was important in providing rind penetrometer resistance, stalk lodging resistance, and ECB resistance.

More recently, four maize inbred lines were selected for their consistent resistance (EP39 and A509) or susceptibility (EP47 and EP42) to corn borers across multiple evaluations [63,64]. The resistant group showed higher total cell wall material in the pith, particularly those components abundant in secondary walls (glucose, xylose, and lignin), suggesting that cell wall thickness may be an important barrier that corn borers must mechanically rupture in order to gain access to the cytoplasmic nutrients contained within the pith tissues [32].

Multiple regression equations to predict ECB damage were based on the concentration of cell wall constituents in most of the previous studies. This approach is effective when the independent variables are not highly correlated. Whenever independent variables are highly collinear, their relative importance cannot be determined using either ordinary least-squares or biased regression methods since the data are inadequate for this purpose [65]. Cardinal and coauthors [66] found that the cell wall components were highly correlated in a recombinant inbred line population (RIL) obtained from the cross of maize inbred lines B73 and B52. Therefore, the authors used a direct approach: the comparisons of quantitative trait loci (QTL) detected for cell wall components and ECB tunneling in the same maize population. NDF, ADF, and ADL levels were analyzed in stalk and leaf-sheath. The results showed that most of the QTL for ECB resistance (10/13) were at QTL positions for one or more cell wall components. Seven of the ten QTL associated with both traits contributed to the negative correlations observed between these traits, while relatively few QTL effects were positively correlated. This suggests that although cell wall components contribute to ECB resistance in this population, other mechanisms and likewise other genes are involved [16].

Genotypic correlations between ECB stalk tunnel and cell wall components were not significant in RIL population developed from the cross of inbred lines B73 and DE811. Nevertheless, clustering of QTL for ECB damage and cell wall components was observed and negative genotypic correlations between both traits were observed at some loci. So, ECB resistance may be associated with a subset of the QTL observed for cell wall components and ADF in particular [17]. Comparing both studies, only three of the nine QTL for ECB resistance in the RILs of B73 × B52 were linked (within 25 cM) to QTL in the RILs of B73 × DE811 [16,18], what showing once again that the relationship found seems to be depend on the genetic materials assessed.

In Spain and other Mediterranean countries, a major insect pest of maize is the Mediterranean corn borer (MCB), *Sesamia nonagrioides* Lefèbvre [67,68]. Like other borers the main importance resides in the second generation larvae that feed on the pith during plant development causing weakening and lodging of the maize plants. Up to 95% of plants may be damaged and yield losses may reach 30% [69,70]. Three QTLs for MCB stalk tunneling detected in the RIL population EP42 × EP39 by Ordás and coauthors [71] were close to QTLs for stalk strength or cell wall constituents identified in various studies, suggesting that genes involved in the synthesis of cell wall compounds could be good candidates for resistance to corn borers. Two QTLs for MCB resistance were found in the RIL population B73 × Mo17 [72]. The QTL for stalk tunneling in the region of bin 9.04 overlapped with the stalk tunneling QTL detected in crosses of B73 with B52, De811 and Mo47, which are three inbred lines derived from diverse genetic backgrounds [73]. The region was also associated with leaf sheath and stalk fiber and, moreover, the allele associated with highest fiber was associated with lowest ECB tunneling [16,17].

Southwestern corn borer (SWCB), *Diatraea grandiosella* Dyar. and Fall armyworm (FAW), *Spodoptera frugiperda* J.E. Smith, are additional pests of maize. These insects attack plants in both vegetative and reproductive stages. In relation with the cell wall composition, Hedin and coauthors [22] demonstrated that resistant inbreds to SWCB have higher hemicellulose and crude fiber content than susceptible inbred lines, and moreover, crude fiber was negatively correlated with larval feeding damage. Additionally, the relationship between hemicellulose and FAW resistance was also observed [23,24]. Subsequent research showed that leaves of resistant hybrids to FAW presented higher amounts of hemicellulose than leaves of susceptible genotypes [25]. Hemicellulose and protein levels appear to be associated with FAW resistance, but not with SWCB resistance. Instead, relative amounts of cellulose were negatively correlated with leaf feeding by both insects. Amounts of ash, fat, and lignin did not appear to be associated with resistance to these pests [25].

The role of the cell wall polysaccharides in disease resistance has been scarcely studied. Mideros and coauthors (2012) [44] investigated the relation between *Aspergillus flavus* Link colonization and different kernel traits in a set of 19 inbred lines. *A. flavus* is the most common causal agent of aflatoxin contamination, aflatoxin B1 being the most potent naturally occurring carcinogen known [74]. They found significant correlations of aflatoxin content with fiber, ash, carbohydrates, and seed weight. Fiber and ash content may correlate with the thickness of pericarp and aleurone layers, reflecting barriers to fungal entrance.

Of the three rust pathogens that occur on corn worldwide, southern rust of corn (SRC), caused by *Puccinia* polysora Underw., has been reported as the most destructive [75]. Higher ADF and NDF contents were found in the leaves of a resistant inbred line to *P. polysora* than in a susceptible, though note that only two genotypes were analyzed [46].

*Fusarium graminearum* Schwabe is one of the most important plant pathogens infecting several cereal species including maize, wheat, and barley, in cold and temperate regions [76,77]. The major economic importance comes from the contamination of grains with mycotoxyns, mainly zearalenone and deoxynivalenol (DON). In maize, both the ear and the stalk can be infected [76,78]. Ester-bound ferulic acid decreased in the silks tissues after inoculation with *Fusarium graninearum*, probably due to degradation of hemicellulose by hydrolytic enzymes produced by the fungus [42]. Differences in the release of ferulates in resistant and susceptible genotypes suggest the influence of hemicelluloses in *F. graminearum* resistance [42].

## 3. Cell Wall Bound Hydroxycinnamates

The most common hydroxycinnamates found in a wide range of grasses are ferulates (FA) and *p*-coumarates (pCA). Cell-wall hydroxycinnamates are derived from the phenylpropanoid pathway, which originates from phenylalanine and tyrosine. Though structurally related, they seem to have different functional roles within the cell wall. FA is the major hydroxycinnamate derivative in young grass cell walls. Maize cell walls can contain up to 5% ferulate monomers plus dimers [79]. Ferulic acid is primarily esterified to arabinosyl residues of arabinoxylan chains, and feruloylated arabinoxylans are later cross-linked to G units of lignins via ether bonds. *p*-Coumaric acid is mainly esterified to the γ-position of the side chains of S lignin units [80,81] and lignified maize cell walls can contain up to 3% *p*-coumarate [79].

It is suspected that FA is a very important components not only for the biology of the cell wall, but also for its structure because they can be coupled by peroxidase- or laccase-mediated oxidative coupling to form a variety of dimers, cross-linking polysaccharide chains [1,82,83]. In addition, the ability of ferulic acid to participate in ester linkages and phenol coupling reactions confers it with the ability to covalently attach polysaccharide with lignin. The resultant product is a ferulate-polysaccharide-lignin complex bonded through ester-ether linkages [8,84]. Thus, FA deposition in the wall may not only lead to cell wall cross-linking during plant growth and development, but may also regulate the non-random pattern of lignin formation within the wall [9,85,86]. On the other hand, the role of pCA in cell wall matrices is not fully understood. It appears the majority of pCA remains unincorporated other than its attachment to monolignols via an ester linkage. Therefore, pCA does not function as a cross-linking agent between wall matrix polymers, at least between different lignin polymers or between lignin and polysaccharides. It has been suggested that pCA may function as radical transfer agent to aid in the formation of sinapyl alcohol and lignin radicals [87,88]. There are reports of pCA being ester linked to arabinoxylans just as for FA, however this level is much lower (1:15) compared to FA [89]. Incorporation of pCA into grass walls appears to be primarily associated with the lignin fraction. Several studies have shown a positive relationship between pCA incorporation into cell wall matrices and levels of lignin formation [90–92].

Accumulation of FA follows the elongation pattern, with up to 70% of alkali-labile ferulate deposition that occurs during secondary wall formation and lignification [93]. Deposition of esterified and etherified ferulate derivatives continue at a high rate after cell elongation has ceased [94]. FA content plateaus as the walls mature [90]. The accumulation of pCA is a relevant indicator of lignin deposition [95,96]. In addition, it indicates a higher proportion of secondary wall in tissue [79].

Although pCA also form cell wall cross-links by the formation of radically or photochemically formed dimers, ferulate derivatives are the quantitatively most important cross-links in the plant cell wall. The first FA dimer (DFA) discovered was the 5-5′-dehydrodimer, isolated after alkaline hydrolysis of the cell wall polysaccharides [97]. Since then, a number of dehydrodimers have been identified and characterized in different grass species. The major forms are: 5-5′-DFA: (*E*,*E*)-4-4′-dehydroxy-5-5′-dimethoxy-3′-bicinnamic acid; 8-0-4′-DFA: (*Z*)-β-4-hydroxy-3-methoxycinnamic acid; 8-5′-DFA: (*E*-*E*)-4-4′-dihydroxy-3,5′-dimethoxy-β,3′-bicinnamic acid; 8-5′-C (benzofuran form): *trans*-5-((*E*)-2-carboxyvinyl))-2-(4-hydroxy-3-methoxyphenyl)-7-methoxy-2,3- dihydrobenzofuran-3-carboxylic acid, and 8-8-DFA (4,4′-dihydroxy-3,3′-dimethoxy-β,β′-bicinnamic acid) [98]. FA and DFAs contents have been estimated to be 25 g kg^−1^ in maize stems [99]. More recently, specific ferulate dehydrotrimers and tetramers have been isolated and identified in a variety of plant tissues [100–103]. Formation of intramolecular versus intermolecular polysaccharide crosslinks is a key question to be answered in the future.

Thus, hydroxycinnamate mediated cross-links are able to connect cell wall polymers, especially polysaccharides to each other but also to lignin and to proteins [9,104]. Plant cell wall cross-linking strengthens the cell wall [105] and is involved in the regulation of the cell wall extensibility and cessation of elongation processes [106–109]. Bonding contributes, as many studies have shown, to the low digestibility of most grass species even by ruminants and, in part, to the recalcitrance of grass tissues to direct degradation by simple mixtures of cellulases and xylanases [56,79,110–115].

There is some minor chemical disruption of the plant cell wall attending to the insect attack, for instance, majority of the plant cell wall is indigestible to lepidopteran [51,116]. Some studies with insects used cellulose as an indigestible nutrient diluter [117,118], but if the effect of the cell wall is that of a barrier to the easily digested contents, then the arrangement of the cell wall components may be more important than the absolute amount of cellulose, hemicellulose, *etc*. Plant nitrogen is a major factor contributing to larval growth [119], however, it does not matter how much nitrogen the plant contains if it is inaccessible. We conclude, once again, that the cell wall matrix appears to be a barrier that insects must mechanically rupture to assimilate nutrients. In accordance with this idea, the link between cell wall cross-linking and the insect resistance was first suggested by Fry (1986) [120]. Since then, various studies have pointed out the role of hydroxycinnamates in pests and diseases resistance [19,27,32,40]. A working theory has proposed that susceptibility of maize to pests and diseases depends on these hydroxycinnamates contents [121,122].

Concerning to ECB resistance, increased levels of FA monomer have been quantified in epidermal cell walls of leaves in maize resistant inbred lines [20], while the content of FA dimers in leaves was negatively correlated across genotypes with the leaf damage [21]. The mechanism of resistance suggests early cell wall fortification through phenolic cross-linking of hemicellulose by DFAs, increasing the hardness of the leaf tissue. Additional evidence was provided by DFAs increases in diverse feeding tissues over cycles of selection to ECB resistance in the Iowa Stiff Stalk Synthetic BS9 [19]. In addition, both DFA and pCA were negatively related with damage parameters in the rind, node and pith [19].

The related insect species SWCB and sugarcane borer (SCB), *Diatraea saccharalis* (Fabricius), are among the most important lepidopteran pests affecting maize production in Central and South America [123]. Later generations damage the plant mainly by stalk boring and tunneling, resulting in indirect yield losses. Resistance to these borers could be sensitive to early cell wall fortification by phenolic cross-linking [26]. The study showed that both cell wall bound pCA and FA were significantly and negatively correlated to leaf feeding damage. Significant negative correlations were also found between DFA content and leaf feeding damage by both insects.

Multiple studies with maize genotypes that differ in their resistance to the MCB noted the role of hydroxycinnamates in the resistance to this borer pest [27–30,32]. Monomers, pCA and FA, and diverse isomers of DFA, were identified in different feeding tissues. The amount of all these compounds in the pith was correlated with the resistance level in the genotypes, with the resistant inbreds having the highest concentrations [27]. Negative correlations were observed between the larvae weight and the DFAs concentration in the leaf-sheath [29]. Subsequent investigations reinforced the effect of hydroxycinnamates in the borer resistance. Higher concentrations of total DFAs were associated with shorter tunnel length and lower numbers of larvae per stem over cycles of selection to MCB resistance in maize synthetic EPS12 [30]. Barros-Rios and coworkers (2011) found that the stem tunneling by ECB and MCB was negatively correlated with total DFAs, 8-5′-DFA and pCA esters [32]. Additionally, it was demonstrated that the DFAs in the pith of the basal section of the maize internode could facilitate MCB larval feeding inside the stem in particular synthetic varieties [28]. Nowadays, Santiago and coworkers has divided the research topic in two branches: evaluation of corn borer resistance in a divergent selection program for DFAs concentration, and study of the variation in the hydroxycinnamates contents after borer attack. Maize populations selected for the highest total DFAs (third cycle of selection for high DFAs (CH3)) had 31% higher total DFAs than maize populations selected for the lowest total DFAs (third cycle of selection for low DFAs (CL3)) [124]. The gain indicates that DFA deposition in maize pith was modified during the selection program and provides unique plant material to validate the genes potentially involved in the FA dimerization. Furthermore, the study demonstrates the powerful role of polysaccharide-polysaccharide cross-linking trough ester-linked DFAs as a natural resistance mechanism of maize against corn borers (damage by stem tunneling was 29% lower in C3H than in C3L) [124]. On the other hand, Rodríguez and coauthors [31] reported that the maize plants respond to the attack of MCB activating genes involved in the cell-wall organization, and moreover, the inbred line PB130 increase twice its DFAs concentration after MCB attack. Current investigations would confirm these motivating findings.

The maize weevil (MW), *Sitophilus zeamais* Motsch., is a destructive insect feeding on stored maize throughout the world [125]. Grain weight losses could range from 10% to 40% during storage [126]. Grain of MW resistant varieties has been characterized attending to the mechanical fortification of the pericarp cell wall. The principal phytochemicals involved in the mechanical fortification are FA, pCA [33], several isomers of DFAs, total phenolic acids, and structural proteins like a hydroxyproline-rich glycoprotein (HRGPs) [34–36]. Strong negative correlations with susceptibility parameters and a positive correlation with grain hardness were found for simple phenolic acids, DFAs, and HRGPs. In addition, in a more recent study the authors identified nine regions common between QTLs associated with MW susceptibility and cell wall bound compounds [37].

There is some evidence in the literature that FA and DFAs are also involved in plant protection against pathogen invasion [40,47]. Much the research attending to the relationship cell wall components and pathogens focuses in fungi. Maydis leaf blight (MLB), *Helminthosporium maydis* Y. Nisik and C. Miyake is the most serious disease in warm and wet temperate and tropical areas, where yield losses close to 70% have been reported. Lyons *et al*. (1993) [47] investigated the distribution of phenylpropanoids during disease development in resistant and susceptible maize cultivars to MLB and reported greater levels of FA and pCA produced in the resistant cultivar.

Structural hydroxycinnamates have been associated with resistance to *Fusarium* diseases in kernel and stalk pith tissues [38,40,41]. Higher contents of pericarp DFAs might act as a preformed structural barrier restricting fungal infection and mycelial progress from diseased to pericarp-intact neighboring kernels [40], while could function in the pith tissues as preformed resistance barriers prior to infection [38]. Low contents of DFAs in the early stages of infection would leave time for the pathogen to develop abundantly and to produce toxins, breaking a possible resistance response and leading to typical symptoms of disease [38]. The role of hydroxycinnamates in the infection of maize silks by *F. graminearum* was also investigated [42]. Although significant changes in the concentration of hydroxycinnamates were observed after inoculation, no role of these compounds as mechanism of resistance was observed in this tissue. As previously noted differential decrease of FA in resistant and susceptible genotypes suggests a possible role of hemicelluloses in the *F. graminearum* resistance in the silks [42].

Likewise, in warmer regions, Fusarium ear rot is prevalent and is the result of kernel infection by *Fusarium verticillioides* (Saccardo) Nirenberg. It not only reduces yield but also contaminates the infected kernels with mycotoxins, mainly fumonisins (FB1, FB2, and FB3). A recent paper reported on the possible role of DFAs in the genotypic resistance to *Fusarium veticillioides*[43]. The contents of the hydroxycinnamates detected in the pericarp of several maize genotypes suggest their contribution to kernel resistance to *F. verticillioides*[43]. In these experiments, resistant genotypes contained about a 3-fold higher level of total DFAs than susceptible ones. A stepwise regression model suggests that the increase of all individual DFA species contributed to limit fumonisin accumulation and mycelial progress of *F. verticillioides*. Crosslinking cell wall polysaccharides results in pericarp hardness [127]. Cell wall DFAs might also have a direct inhibitory effect on mycotoxin production after being released by fungal esterases and other extracellular enzymes during infection of *F. verticillioides*. The 8-5′-C DFA, the major DFA detected in the pericarp of the genotypes, showed *in vitro* to be as effective as FA to inhibit the biosynthesis of trichothecenes by *F. graminearum*[128].

Less work has been carried out quantifying the dimer content of different cell types, or if these different DFAs have different physiological roles. As better detection methods are developed, the extensive network of cross-links involving these phenolic compounds will be better conceived.

## 4. Lignin

Lignin is the second most abundant polymer found in nature after cellulose. Among the many roles lignin plays in plant growth and development are those providing structural support for land plants and as a resistance mechanism to biotic and abiotic stresses. Lignin is an end product of the phenylpropanoid pathway and a heteropolymer of three hydroxycinnamyl alcohol monomers or monolignols: *p-*coumaryl alcohol, coniferyl alcohol, and sinapyl alcohol [129]. The intermediate products of the monolignol biosynthetic pathway serve as precursors of hydroxycinnamates and other phenolic compounds. Both monolignols and their precursors are synthesized in the cytosol (endoplasmic reticulum) and later transported to the wall where lignin is deposited. Because of monolignols are relatively toxic to the cell, it has been suggested that the exported and storage forms are monolignol-glucosides. Once localized in the wall, monolignols are catalyzed by peroxidase and/or laccase to form three phenylpropanoid units: *p*-hydroxyphenyl (H), guaiacyl (G), and syringyl (S). The deposition of each lignin type in plant tissues is spatially and temporally controlled, first being deposited H units, followed by G units, and finally S units [130]. It has been found that mature maize stalks contain 4% H, 35% G, and 61% S. In general, the S/G ratio increases as the plant matures and the lack of S units creates a more condensed lignin (*i.e.*, the prevalence of C–C linkages and low methoxylation stage). The ratio between S and G units (S/G) are commonly between 1.02 and 1.61 for maize stalks and 0.20 and 0.55 for leaves. The maize the brown midrib mutation results in a reduction of lignin content (≈35%) and a variation on lignin composition with S/G ratios between 0.34 and 1.95 [131,132].

Tissue toughness is one of the key factors that regulate herbivore damage in plants [133,134] and the tougher the tissue, the greater lignin content. Tissues with high lignin concentration and otherwise cross-linked, are less palatable than tissues with low lignin content. Most research carried out to evaluate the effect of lignin on maize resistance to insects based on the analysis of forage fiber fractions (ADF, NDF, and ADL) has been commented on Section 2.

Increased lignin deposition might have additional negative effects on insect fitness because phenoloxidase enzymes are involved in lignin polymerization but also in generation of toxic by-products as reactive oxygen species, quinones and peroxides [135,136]. It is known that several of the defense-related maize metabolites are biosynthetically related. Maysin, chlorogenic acid and phenolic acids originate from the phenylpropanoid pathway [137].

Cell wall lignification may be also induced when plants are wounded or become infected by pathogens or insects, suggesting that lignin may act as a chemical or physical barrier to protect the rest of the plant cells from further damage [54,138,139]. This lignin induced as a response to pathogen attack is considered “stress lignin”, and has higher amount of the simpler (H) lignin subunits [140,141]. Ride [142] pointed out that lignification might hinder fungal growth through plant tissue in several ways. First, increased cell wall lignification and stiffness at the point of fungal attack may make walls more resistant to mechanical penetration and render it resistant to dissolution by fungal enzymes. Second, lignification of walls may restrict diffusion of enzymes and toxins from the fungus to the host and of water and nutrients from the host to the fungus, in essence starving the fungus. Third, phenolic precursors of lignin and free radicals produced during polymerization may inactivate fungal membranes, enzymes, toxins, and elicitors. Accordingly, Lyons and coauthors [47] attributed the increased lignin deposition to the infection of the leaf corn fungus *H. maydis*. In addition, Liu *et al*. [39] suggested that potassium chloride stimulates the rapid expression of PAL, tyrosine ammonia-lyase (TAL), cinnamyl alcohol dehydrogenase (CAD), and phenoloxidase activities in both resistant and susceptible maize genotypes after pathogen inoculation, increasing the induced lignin content, and enhancing the resistance of maize to *Fusarium* stalk rot. The lignification process in maize cobs from resistant and susceptible inbred lines to the pathogenic fungus *A. flavus* was studied by Spangler [45]. Lignin extracted using thioacidolysis revealed that resistant lines contained higher lignin than susceptible ones. Regarding to lignin composition, resistant inbreds had two times more G subunits than susceptible ones. However, real-time quantitative PCR showed no significant differences between genotypes for the transcription levels of the last enzyme of the lignin biosynthetic pathway caffeoyl-coA-Omethyltransferase 1 (CCoAOMT1).

In brief, increased toughness and concentration of toxic by-products of lignin synthesis (phenoloxidase activity) seem to be the most likely mechanisms for the lignin associated protection of plant tissues from pests and diseases.

## 5. Conclusions

Several cell wall components show a determinative role in maize resistance to pest and diseases. However, defense mechanisms are very complex and vary among the same plant species, different tissues or even the same tissue at different developmental stages. Probably we do not get a clear relationship with an overall determination of cell wall components in the whole plant of a single genotype, thus, it is important to note that the role of the cell wall components needs to be tested in diverse genotypes and specific tissues where the feeding or attacking by the pathogen takes place. Diverse studies attending to this genotype-tissue-specific content have been noted in the current review.

Understanding the role of cell wall constituents as defense mechanisms may allow modifications of crops to withstand pests and diseases. Nevertheless, we have to take into account that this defense is interconnected with other biological processes, many genes and pathways that work in the insect attack are also involved in disease defense or in responses to other stress agents. Modifications on the cell wall structure and composition should be evaluated on various pest and disease combinations in order to assess the impact on crop agronomic fitness.

## Figures and Tables

**Table 1:** Summary of literature attending to the role of the cell wall composition on maize resistance to pests and diseases.

Pest or disease	Tissue/organ	Cell wall component	References
*Ostrinia nubilalis* (ECB)	whorl tissue and leaf sheath, leaf blade and stalk	NDF, ADF and lignin	[11,12]
ECB	stalks and leaf sheaths	crude fiber, cellulose, hemicellulose, NDF, ADF, NDF adjusted by ADL, ADL, lignin and silica	[13–18]
ECB	immature and mature leaf blade and leaf sheath, rind, node, and pith	crude fibre and hydroxycinnamates	[19–21]
*Diatraea grandiosella* (SWCB) and *Spodoptera frugiperda* (FAW)	whorl leaves and leaves	hemicellulose, cellulose, lignin	[22–25]
SWCB and *Diatraea saccharalis* (SCB)	leaves	hydroxycinnamates	[26]
*Sesamia nonagrioides* (MCB)	stalk, pith, and leaf-sheaths	hydroxycinnamates	[27–31]
MCB and ECB	pith and rind	cell wall polyssacharides, lignin and hydroxycinnamates	[32]
*Sithophilus zeamais* (MW)	grain and pericarp	protein and hydroxycinnamates	[33–37]
*Fusarium graminearum*	pith	Hydroxycinnamates lignin	[38,39]
*F. graminearum*	grain, pericarp and aleurone	hydroxycinnamates	[40,41]
*F. graminearum*	silks	hemicellulose	[42]
*Fusarium verticillioides*	pericarp	hydroxycinnamates	[43]
*Aspergillus flavus*	grain	fiber and carbohydrates	[44]
*A. flavus*	cob	lignin content and composition	[45]
*Puccinia polysora*	leaves	NDF and ADF	[46]
*Helminthosporium maydis*	leaves	hydroxycinnamates	[47]

## Abbreviations

FA: Ferulate
pCA: *p*-Coumarate
DFA: Diferulates
NDF: neutral detergent fiber
ADF: acid detergent fiber
ADL: acid detergent lignin
HRGPs: hydroxyproline-rich glycoprotein
ECB: European corn borer
SWCB: Southwestern corn borer
SCB: Sugarcane borer
MCB: Mediterranean corn borer
MW: Maize weevil
SRC: Southern rust of corn
MLB: Maydis leaf blight
